# Efferocytosis in heart failure: Mechanisms, dysregulation and therapeutic potential

**DOI:** 10.7150/thno.132477

**Published:** 2026-05-01

**Authors:** Otega Oviri, Shijie Liu, Hongye Huang, Xiaoyuan Bai, Jingke Yao, Zufeng Ding

**Affiliations:** Department of Biology, Georgia State University, Atlanta, GA 30303, USA.

**Keywords:** heart failure, cardiac function, efferocytosis, phagocytes, macrophages

## Abstract

Heart failure (HF) remains one of the leading health problems globally. It is often characterized by unresolved inflammation, adverse cardiac remodeling, and tissue damage. Efferocytosis, the process by which phagocytes clear apoptotic cells, is a key process that helps to resolve inflammation and maintain tissue homeostasis. In the healthy heart, efferocytosis ensures the proper removal of apoptotic cardiac cells (e.g., cardiac fibroblasts and cardiomyocytes) and promotes inflammation resolution and secretion of anti-inflammation cytokines. However, emerging evidence suggests that this process is often impaired in patients with heart failure, and as a result leads to chronic inflammation, secondary necrosis, and tissue scarring. This review explores the cellular and molecular processes guiding efferocytosis and how its dysregulation potentially contributes to advanced heart failure. It also explores how factors like macrophage and endothelial cell phenotypes, lipid mediators, and nuclear transcription factors influence the efficiency of efferocytosis in the myocardium. By synthesizing current findings, we propose that restoring efficient efferocytosis in the failing heart may present a promising therapeutic approach to resolving inflammation and improving the outcomes of patients with heart failure.

## Introduction

Heart Failure (HF) is a serious health condition that makes it very difficult for the heart to pump blood to the body for its metabolic needs as well as properly manage the return of blood to the heart 1. It affects over 64 million individuals worldwide 1. According to the Heart Failure Society of America (HFSA) 2024 report, there are about 6.7 million people in America alone over the age of 20 who are currently living with HF 2. This number has been predicted to rise to approximately 11.4 million people by 2050 2. Based on this definition, HF is majorly classified into two major types based on how the heart pumps enough blood out and also how fast it fills with blood in normal conditions 3. When the heart can’t pump enough blood out, it is referred to as HF with Reduced Ejection Fraction (HFrEF); and when the heart muscles are stiff and can’t let blood fill in properly then it is known as HF with Preserved Ejection Fraction (HFpEF) 3. However, the use of this broad classification for HF might not be sufficient enough based on studies for patients with HF conditions, which are mid-range and some with also a recovered ejection fraction 3. Importantly, HFrEF and HFpEF differ not only in their hemodynamic characteristics but also in their underlying inflammatory and immunological profiles 3. HFrEF is typically associated with ischemia-driven cardiomyocyte loss, resulting in a high burden of apoptotic cells that require efficient clearance. In contrast, HFpEF is characterized by chronic low-grade inflammation, endothelial dysfunction, and progressive fibrosis 4. These differences suggest that efferocytosis may play distinct roles across HF phenotypes, influencing both inflammatory resolution and cardiac remodeling. The presence of HF in humans normally presents symptoms such as consistent tiredness, swelling of the ankles, and shortness of breaths with closely associated signs which may include abnormal sounds in the lungs, pressure in the jugular veins all of which leads to a certain kind of structural and functional abnormality of the heart 1. The leading causes of HF are ischemia, diabetes, and high blood pressure 1. Myocardial ischemia accounts for about 70% of HF and contributes a lot to the progression of HFrEF and HFpEF 3. High blood pressure plays another major role in the development of HFpEF, especially a type of hypertension-induced left ventricular hypertrophy (LVH) that basically means thickening of the heart muscle 3. Additionally, according to previous research 5, Type 2 diabetes is also strongly linked as a risk for both HFrEF and HFpEF. Some other possible causes may include diseases that involve the heart valve, toxins such as alcohol and cytotoxic drugs, irregular heart rhythms, and inherited heart diseases 1.

While the major causes of heart failure (e.g., atherosclerosis, hypertension and diabetes) are well recognized and understood 3, 5, 6, recent research studies have begun to underline the significant role that immune dysregulation plays in the development and progression of heart failure 5. To be specific, the body’s immune cells response, including macrophage activation, cytokine release, and failure to clear apoptotic cardiac cells, leads to chronic inflammation and adverse remodeling of the heart muscle 3. Macrophages, play a central role in HF development because they can either promote inflammation (M1 type) or resolve inflammation and promote tissue repair (M2 type) 7. In HF, an imbalance between these macrophage phenotypes can lead to persistent inflammation, damage to tissue and, fibrosis 7. This emerging view strongly suggests that HF is not only a result of traditional risk factors earlier outlined in this review but also a result of immune system dysregulation. Consequently, immune regulation has become a significant point of interest, with recent in-depth studies and analysis showing the importance of immune regulation in HF 8. This growing body of research has begun to open new areas for targeting immune regulation as a potential treatment strategy in the management of HF. In this review, we will mainly focus on efferocytosis in heart failure and summarize its mechanisms, dysregulation and therapeutic potential.

## Efferocytosis and its Role in Immune Homeostasis

Efferocytosis is a terminology that is derived from the Latin word “*effero*” which means “*to bury*” 9. It is a process by which professional phagocytic cells like macrophages and dendritic cells, remove apoptotic cells from tissues in a very coordinated manner 9. This phagocytic ‘burial’ activity of apoptotic cells was conceptualized by scientists and subsequently coined in the year 2006 by Peter Henson and his research team 9. It is a removal mechanism that is very essential for maintaining tissue homeostasis and immune regulation in the human body 9. In multicellular organisms, millions of cells undergo programmed cell death daily and need to be replaced for normal tissue homeostasis 10. The efficient removal of these dying or dead cells is critical to avoiding secondary necrosis, which is a form of uncontrolled cell death that can trigger inflammation 10. Although, in contrast to professional phagocytosis, efferocytosis is characterized by a complete absence of inflammatory and immunological activity, which ensures the removal of apoptotic cells without activating any sort of inflammatory response 10.

Efferocytosis comprises of three main processes which are recognition of the apoptotic cells, engulfing them, and subsequently degrading the dead cells 11. The proper phagocytic removal of these cells requires a cohesive and precise assembly of different associated molecules and key changes to some cell machinery 11. There are three key signals in efferocytosis, which are the ‘find me signals’, ‘eat me signals’ and ‘don’t eat me signals’ 11. These signals, which lead the process of efferocytosis have all been shown to be important for this mechanism to take place 11. At the beginning of efferocytosis, apoptotic cells initiate a molecular cascade by releasing ‘find me signals’ which include sphingosine -1- phosphate (S1P), lysophosphatidylcholine (LPC), adenosine triphosphate (ATP), and CXC3CL1 chemokine ligand 1 (CXC3L1, commonly referred to as fractalkine) which serve as the first recruitment mechanism for phagocytic cells to the area of apoptotic cell death 12. Upon the arrival of phagocytes, they recognize the ‘eat me signals’, the most notable of which is phosphatidylserine (PtdSer) which is normally displayed on the surface of cells after apoptosis 11. Many receptors that are found on the phagocytes like Mer tyrosine kinase (MerTK), T cell immunoglobulin, mucin-domain containing 4 (Tim-4), and integrins, recognize these signals 11. However, some of them, like MerTK carry out this recognition by coordinating other bridging molecules such as Gas6 and MFG-E8. In contrast, viable cells would normally express ‘don’t eat me signals’ like CD47, to ensure that efferocytosis is specific and prevent misengulfment 11.

Efferocytosis has significant implications in resolving inflammations and goes way beyond normal cell clearance 11. This is because, during the process of ingestion, phagocytes normally undergo a functional configuration, which leads to the emission of anti-inflammatory cytokines such as IL-10 and TGF-β, whilst also downregulating the production of pro-inflammatory cytokines such as TNF-α and IL-1β. These simultaneous functional changes are very instrumental in bringing about the process of tissue restoration and remodeling, as well as reestablishing a state of immune equilibrium 13. Efferocytosis also ensures that there is an equilibrium in immunological balance by facilitating the proper clearance of apoptotic cells 11. This process is also crucial in preventing autoimmune responses through the removal of potentially immunogenic self-antigens prior to lymphocyte activation and also contributing to the structural and functional integrity of tissues that produce a lot of cells, like the lung, liver, and heart 11. In these tissues, a large number of cells usually undergo apoptosis daily as a result of mechanical stress (in the heart and lungs), high metabolic activity (in the liver), or even as a result of immune surveillance 14. Efficient efferocytosis ensures that these dead cells are properly cleared without triggering an inflammation making sure that the surrounding tissue structure remains intact. On the contrary, impaired efferocytosis in these tissues can lead to the accumulation of apoptotic cells and hereby induce secondary necrosis and the release of damage-associated molecular patterns (DAMPs), which causes substantial inflammation and tissue damage 11. For instance, in the heart, impaired efferocytosis can lead to tissue scarring and dysfunction of the ventricles 15. In the liver, the failure to clear dead hepatocytes heavily contributes to conditions such as tissue injury and fibrosis 16. Meanwhile, in the lungs, delayed efferocytosis of alveolar cells has been associated with chronic inflammation and acute lung injury 17. This implies that efferocytosis is not just about cell clearance but also serves as a critical mechanism for immune regulation and the integrity of tissue.

In the following sections, we will examine the molecular mechanisms of efferocytosis in detail by focusing on important signaling pathways, receptor-ligand interactions, and intracellular pathways that regulate this important process, and more importantly, how their dysfunction contributes to chronic inflammatory conditions in HF.

## Mechanisms of Efferocytosis

**Definition and cellular players of efferocytosis.** Efferocytosis is the specialized and rapid clearance of apoptotic cells, mainly performed by professional effeocytes of macrophages. Other phagocytes such as dendritic cells 18 and non-professional effeocytes (e.g., epithelial cells, fibroblasts and aortic endothelial cells) can also perform efferocytosis 18. However, macrophages excel at performing high-capacity, rapid, and sustained efferocytosis; in contrast, non-professional effeocytes exhibit slower phagocytic kinetics and lower phagocytic capacity, and often serve as secondary scavengers within specific microenvironments 1,2,18,19. Non-professional phagocytes perform efferocytosis to clear adjacent dying cells, acting as a crucial secondary cleanup system for tissue homeostasis. Neutrophils, however, are not considered professional efferocytes; instead, they can indirectly regulate efferocytosis by releasing mediators that influence macrophage polarization and efferocytic capacity 19. Remarkably, several phagocytes that are not traditionally considered as ‘professional’, such as aortic endothelial cells, epithelial cells and fibroblasts, also have an ability to perform efferocytosis 20. Efferocytosis exhibits distinct mechanisms and biological implications that are different from the traditional phagocytosis. Phagocytosis involves pathogen recognition through pathogen–associated molecular patterns (PAMPs) by phagocytes with Toll-like receptors (TLRs) or other proteins like complement and Fc receptors, especially when the pathogens are tagged for removal. On the contrary, efferocytosis is conducted by certain phagocytic receptors that sense the ‘eat me’ signals which are present on the surface of apoptotic cells mainly via receptors from the family of TIM (T cell immunoglobulin and mucin) and TAM (Tyro3, Axl, Mer) 21. From a physiological perspective, efferocytosis is distinguished by its anti-inflammatory and quiet response, which is very different from the release of inflammatory cytokines during traditional phagocytosis 11. The clearance of apoptotic cells during efferocytosis do not trigger the release of pro-inflammatory cytokines; instead, it brings about the subsequent release of anti-inflammatory cytokines that promote the return to tissue homeostasis. Again, during efferocytosis, morphological modifications are characterized by the dynamic membrane reorganization of the phagocytic cell, resulting in the formation of membrane ruffles that facilitate the engulfment and phagocytic clearance of apoptotic cells 11.

**Major cellular players of efferocytosis.** Macrophages, which are professional phagocytes, are the primary cells in efferocytosis. They are equipped with a wide range of receptors and signaling pathways that allow them to recognize and engulf apoptotic cells efficiently 22. Beyond the clearance of dead cells, macrophages respond to signals in their environment and will normally differentiate into pro-inflammatory or anti-inflammatory macrophage phenotypes based on those signals. This functional phenotypic change significantly impacts the efficiency of efferocytosis 11. Anti-inflammatory macrophages are generally associated with enhanced capacity for efferocytosis as a result of possession of upregulated efferocytotic receptors like MerTK, TIM-4 and integrins, as well as the secretion of anti-inflammatory cytokines such as IL-10 and TGF-β that promote tissue repair 23. On the other hand, pro-inflammatory macrophages tend to express lower levels of efferocytic receptors and secrete pro-inflammatory cytokines such as TNF-α and IL-1β, which can hinder the recognition and engulfment of apoptotic cells 24. For instance, in a study 25, a dominance of M1 macrophages was linked to poor clearance and inflammation in heart disease. This suggests that an inclination of macrophage towards the pro-inflammatory phenotype creates an inflammatory microenvironment that hinders efferocytosis and contribute to the progression of HF. Dendritic cells, which are mainly found in lymphoid tissues, play a unique and important role that comprises the clearance of apoptotic cells and also the presentation of self-antigens under tolerogenic conditions, which is important for preventing autoimmune responses maintaining immune balance 26. Unlike macrophages, dendritic cells do not typically release large amounts of anti-inflammatory cytokines during efferocytosis, instead, their main role is to coordinate adaptive immune responses by presenting of antigens and interacting with T lymphocytes 18. Neutrophils, which also have phagocytic abilities and a short lifespan, also go through apoptosis 11. However, recent findings suggest that under certain inflammatory conditions, neutrophils can participate in a limited form of efferocytosis 27. Even though neutrophils lack certain efferocytic receptors that are typically present in macrophages, they can release molecules that reprogram macrophages towards an anti-inflammatory phenotype (M2) with increasing efferocytic capacity 28. Recent findings have shown that anti-inflammatory (N2) neutrophils can reprogram macrophages to increase their efferocytic capacity. In a recent study by 28, it was demonstrated that macrophages exposed to secreted molecular factors of N2 neutrophils upregulated factors such as CD206, IL-10, and TGF-β, and also increased the expression of receptors and specific surface markers such as MerTK, CD36, CX3CR1, alongside bridging molecules like Gas6 and MFG-E8, which are all important in efferocytosis and the resolution of inflammation.

Macrophages remain the primary efferocytes, equipped with specialized receptors and signaling pathways that enable efficient apoptotic cell clearance and resolution of inflammation 29. Dendritic cells may contribute under specific immunological contexts, particularly in antigen presentation and immune regulation 18. Together, these cell types form a complex and collaborative system that ensures apoptotic cells are cleared efficiently, inflammation is kept in check, and damaged tissue can heal properly.

**Macrophage Heterogeneity in Cardiac Efferocytosis** While macrophages are the primary efferocytes 29, emerging evidence indicates that they are not a uniform population but instead comprise distinct subsets with specialized functions. Macrophages in the heart comprise heterogeneous populations with distinct origins and functions 30. Resident cardiac macrophages, derived from embryonic progenitors, play key roles in maintaining tissue homeostasis and efficient efferocytosis under steady-state conditions 31, 32. In contrast, recruited monocyte-derived macrophages dominate following injury and often exhibit heightened inflammatory responses 33. Emerging evidence suggests that these populations differ in their efferocytic capacity and immunoregulatory functions, with resident macrophages generally displaying enhanced apoptotic cell clearance and pro-resolving phenotypes 34. In heart failure, particularly in HFrEF, the influx of inflammatory monocyte-derived macrophages may impair efficient efferocytosis; whereas in HFpEF, chronic low-grade inflammation and metabolic dysregulation may alter macrophage function and resolution capacity 35. These distinctions underscore the importance of macrophage heterogeneity in shaping cardiac remodeling outcomes.

**Figure 1:**
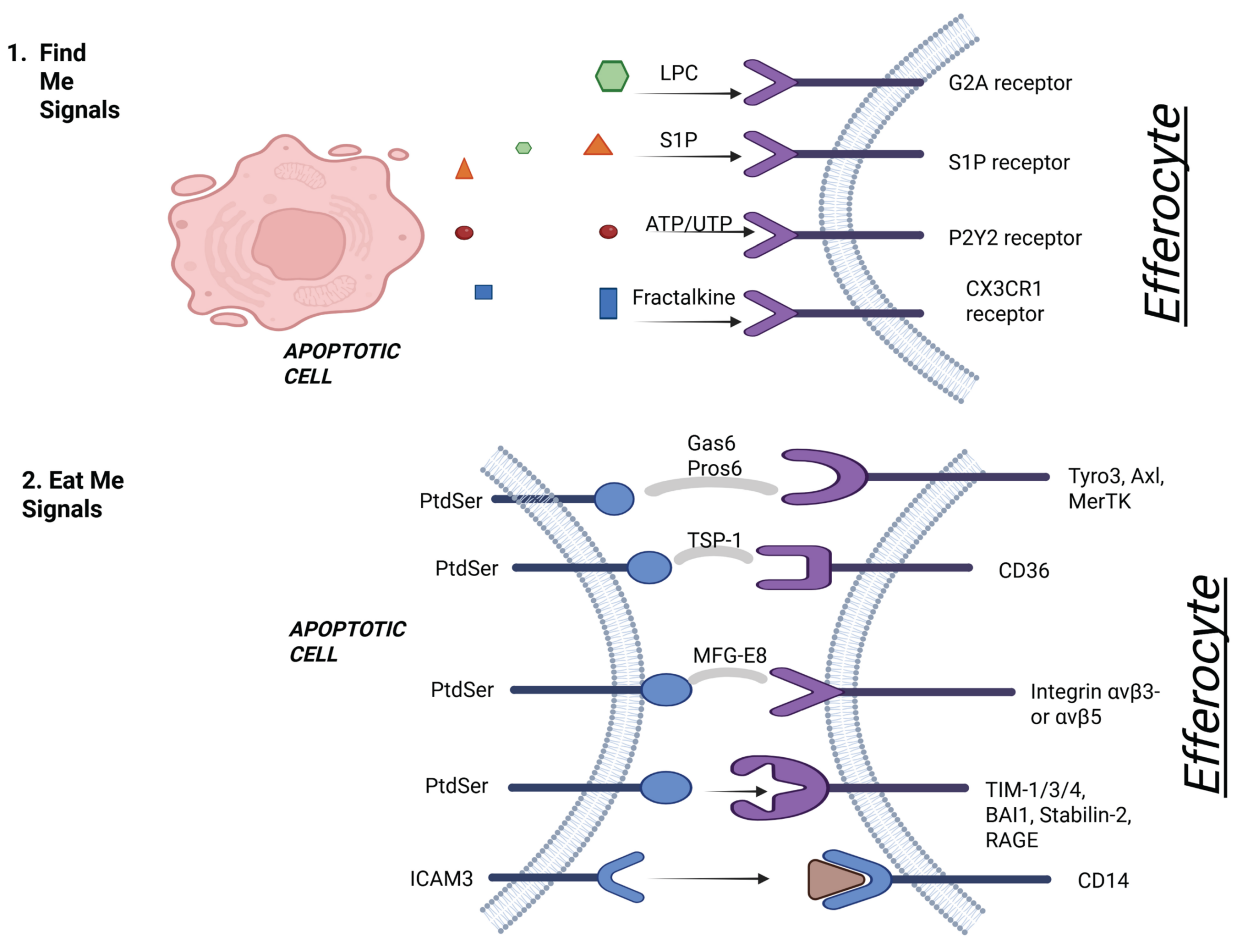
** Schematic general representation of efferocytosis of apoptotic cardiomyocytes by efferocytes.** The process begins with the release of “find-me” signals (e.g., LPC, S1P) from apoptotic cardiomyocytes, which recruit efferocytes to the site of cell death. In the recognition phase, exposed “eat-me” signals such as phosphatidylserine (PtdSer) on apoptotic cells are detected by efferocytic receptors (e.g., MerTK, TIM-4, Stabilin-2), often via bridging molecules like Gas6 and Protein S. In contrast, intact cells expressing “don’t eat-me” signals (e.g., CD47) are spared via their interaction with SIRPα. Engulfment leads to the internalization of apoptotic cells into efferosomes, where they are subsequently degraded. Created with BioRender.com.

**Figure 2:**
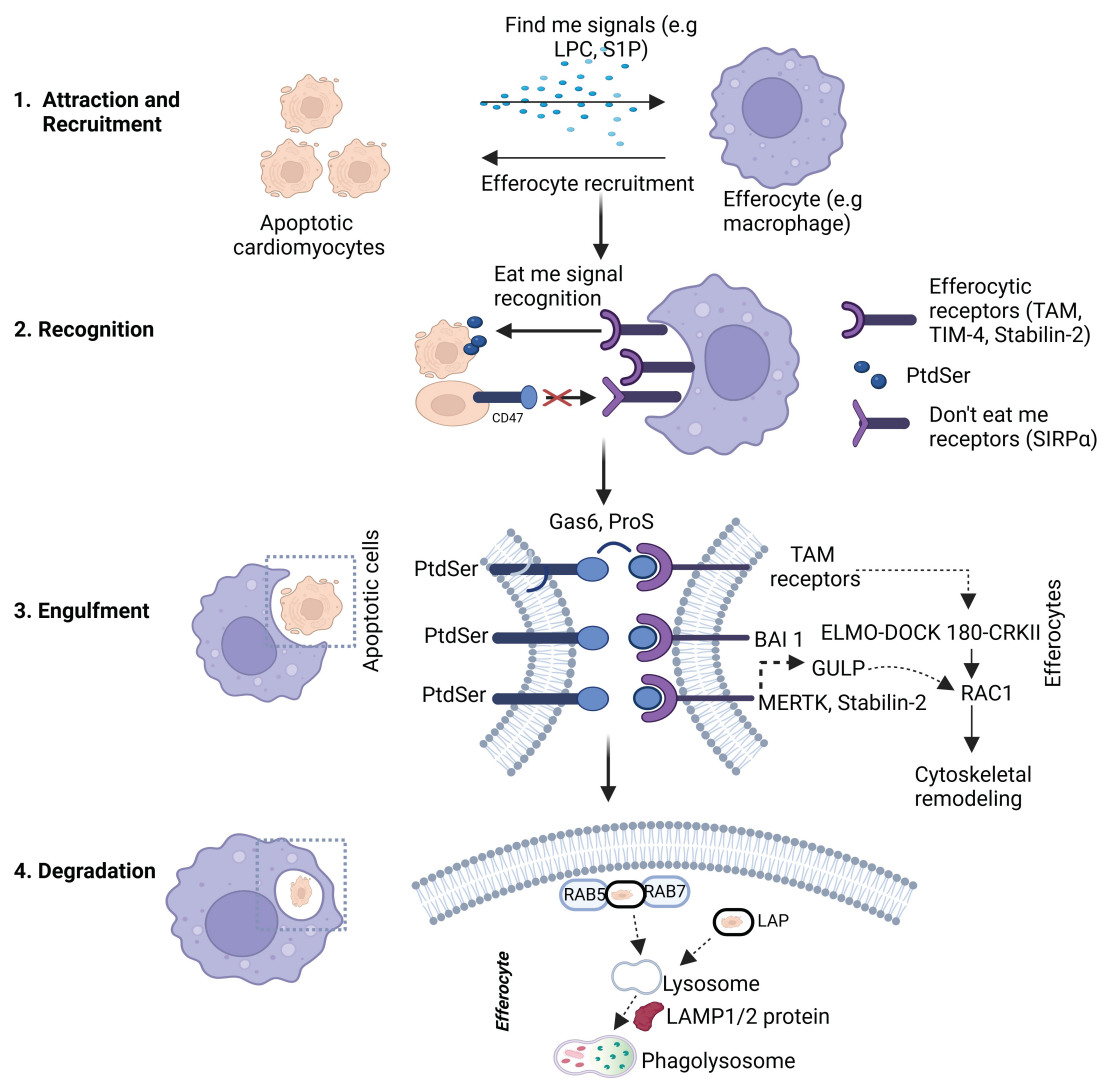
** "Find Me" and "Eat Me" signaling in apoptotic cell recognition.** This figure illustrates the early phases of efferocytosis. In step 1, apoptotic cells release soluble "find me" signals—lysophosphatidylcholine (LPC), sphingosine-1-phosphate (S1P), ATP/UTP, and fractalkine—that bind to corresponding receptors (G2A, S1P receptor, P2Y2, CX3CR1) on efferocytes to facilitate recruitment. In step 2, "eat me" signals—primarily phosphatidylserine (PtdSer)—are exposed on the apoptotic cell surface and interact with various receptors on efferocytes via bridging molecules like Gas6, Protein S, MFG-E8, and TSP-1. These interactions promote apoptotic cell recognition through receptors such as MerTK, Axl, Tyro3, CD36, integrins (αvβ3/αvβ5), TIM family members, Stabilin-2, RAGE, and CD14. Created with BioRender.com.

**Molecular Pathways in Efferocytosis.** Efferocytosis represents a specialized form of phagocytosis that is functionally distinct from classical pathogen phagocytosis and LC3-associated phagocytosis (LAP) 36. While pathogen phagocytosis typically triggers pro-inflammatory responses aimed at host defense, efferocytosis is inherently anti-inflammatory and promotes immune resolution and tissue repair 37. Although LAP shares certain molecular components with efferocytosis, such as LC3 recruitment, it differs in its signaling context and functional outcomes 11. These distinctions are particularly important in the heart, where efficient efferocytosis supports inflammation resolution and limits adverse cardiac remodeling following injury**.** Efferocytosis is a highly coordinated process that comprises of attraction, recognition, internalization (engulfment) and degradation. Each stage is appropriately coordinated by a network of signaling molecules and receptor-ligand interactions that enable the swift and non-inflammatory removal of dying cells. Any change at any point within this process can lead to impaired clearance, immune activation, and chronic inflammation as seen in various conditions like HF and atherosclerosis 11. ***Attraction – “Find me” Signals.*** Apoptotic cells secrete a range of signals to attract nearby phagocytes to the site of death. This is crucial in starting the process of efferocytosis and eventually guiding monocytes, macrophages, and dendritic cells to the site of cell death (**Figure 1** and** Table 1**). ***Recognition – "Eat-Me" and "Don’t-Eat-Me" Signals*.** Once phagocytes reach the site of death, they have to distinguish between apoptotic cells and viable cells. This differentiation is achieved through a balance of “eat me” and “don’t eat me” signals that are displayed on the cell surface 11 (**Figure 2** and** Table 1**).

***“Eat me” Signals.*** During the process of apoptosis, one of the most important “eat me” signal is the external presence of phosphatidylserine (PtdSer) which is flipped from the inner leaflet to the outer leaflet of the apoptotic cell membrane 11. Normally, this phospholipid resides within the inner membrane of normal healthy cells but is moved to the outer membrane in apoptotic cells, resulting from the inactivation of flippases when ATP11 is cleaved and enzymatic activation of scramblases such as Xkr8 as reported previously 38. Although it is important to note that asides from PtdSer, various “eat me” signals have been reported 11. They include oxLDL, ICAM 3, Annexin 1, and calreticulin. More recently, studies have shown that a specific pleioform GADPH also mediates efferocytosis 39. Amongst them all, PtdSer has attracted the significant attention due to its widespread interaction with apoptotic cells and specifically because of its profound inhibitory effect on efferocytosis when its expression is masked 11 (**Figure 1** and** Table 1**).

Phagocytic recognition of PtdSer occurs via two main mechanisms: **1**) Direct efferocytic receptors: Such as BAI1 (brain angiogenesis inhibitor 1), TIM 1/3/4, Stabilin-2 which interact directly with PtdSer on apoptotic cells 40. It has also been reported that the soluble form of RAGE (receptor for advanced glycation end receptors) interacts directly with PtdSer on apoptotic cells 41. ICAM 3 on apoptotic cells have also been demonstrated to have direct interactions with CD14 on phagocytes 42. **2**) Bridging molecule dependent efferocytic receptors: This includes the TAM family of receptors (Tyro3, Axl, and MerTK), which do not directly interact with PtdSer, but rely on bridging ligands such as Gas6 and Protein S. These ligands simultaneously bind to both PtdSer exposed on the apoptotic cells and to the TAM receptors on phagocytes to facilitate the efferocytic uptake process 21, 43. MFG-E8 (Milk fat globule-epidermal growth factor-factor 8) also serves as a functional bridging ligand that interacts with PtdSer present on apoptotic cells and αvβ3- or αvβ5-integrins expressed on phagocytes 44. TSP-1 (Thrombospondin-1) is another ligand which binds to apoptotic cells and coordinates with CD36 on phagocytic cells. This interaction particularly takes place during the clearance of lipid-enriched apoptotic debris and has been implicated in chronic inflammatory conditions such as atherosclerosis 45.

***Don’t Eat Me Signals.*** During efferocytosis, phagocytes have to selectively phagocytose apoptotic cells while avoiding the uptake of healthy and viable cells, even if they display PtdSer on their surface membranes. The cell’s fate as a candidate for efferocytosis or maintaining cellular integrity is determined by the equilibrium of the “eat me signal” and “don’t eat me signal” 42. “Don’t eat me” signals function as critical checkpoints in preventing phagocytic ingestion of healthy and viable cells. They achieve this by either blocking efferocytic receptor activity on phagocytes or antagonizing pro-engulfment signals such as PtdSer presentation 11. Their expression is usually downregulated during apoptosis, hereby tipping the balance toward clearance 38. Although, the prolonged expression of these signals by dying or aberrant cells such as senescent, infected, or tumor cells can hinder normal efferocytotic processes and contribute to chronic inflammation, tissue damage, or even immune evasion by tumor cell 42.

Some “don’t eat me” signal interactions which have been extensively studied including CD47-SIRPα Axis, CD31 and emerging “don’t eat me” signals (**Figure 2**). ***CD47-SIRPα Axis*** – CD47 which is a transmembrane protein found on most nucleated cells, is one of the “don’t eat me signals” that has been frequently studied. It exhibits immunomodulatory properties by binding to a signal regulatory protein called alpha (SIRPα), which is also a receptor present on macrophages and dendritic cells. Upon binding, a series of intracellular events is triggered, which includes the phosphorylation of the SIRPα internal domain and the recruitment of two key enzymes; SHP-1 and SHP-2 phosphatases 46. This ultimately leads to downstream inhibition of the cytoskeletal changes essential for cellular engulfment 46. In a physiological context, this interaction serves as a self-recognition mechanism to prevent indiscriminate phagocytosis. However, the overexpression of CD47 as observed in senescent cells, tumor cells or cells embedded within a pro-inflammatory microenvironment enables these cells to evade immune clearance and contributes to disease persistence 46. Research is currently underway to investigate the therapeutic blocking of CD47-SIRPα as a strategy to revive the phagocytic clearance mechanisms in cancer and cardiovascular disease models 47. ***CD31***, also known as platelet endothelial cell adhesion molecule-1 (PECAM-1), is another inhibitory signal that usually mediates homophilic interactions between live cells. The binding of CD31 to CD31 on cells transmits a negative signal inhibiting phagocytic synapse formation, however, this signal is disrupted during apoptosis following cleavage or conformational changes to CD31, thereby permitting efferocytosis. This mechanism is particularly relevant in vasculature contexts involving endothelial cells and leukocytes as well 42. ***Emerging “don’t eat me” signals.*** Several other molecules have been identified in recent times as potential “don’t eat me” signals or modifiers of efferocytosis. These include CD24, CD200, MHC-1, PD-L1 42. These alternative signals are less characterized in comparison to CD47, although they underscore the evolving complexity of efferocytic regulation and present additional therapeutic targets.

## Mechanism of Engulfment and Degradation of Apoptotic cell

***Engulfment.*** After the detection of apoptotic cells via the exposure of “eat me signals” such as PtdSer, the process of cell engulfment is subsequently activated (**Figure 2** and** Table 1**). This tightly regulated process involves the reorganization of the cytoskeleton, and the formation of a phagosome, which is then followed by the degradation of the apoptotic materials and the reprogramming of the phagocytic cell to be more anti-inflammatory. The efficient implementation of these important steps ensures the proper clearance of dead cells in an anti-inflammatory manner, thereby maintaining the integrity of the tissue 18, 48. This process heavily relies on the activation of a small family of proteins referred to as Rho-family GTPases, especially Rac1, RhoA, and Cdc42 which function as molecular switches to regulate the dynamics of actin 42.

***Rac1 Activation and Actin Polymerization.*** Rac1 functions as an important effector within the intricate process of efferocytosis, and plays a major role in the polymerization of actin during the extension of pseudopodia of the phagocyte. The activation of Rac1 is frequently influenced by the ELMO1-DOCK180-CRKII complex which transfers signals from a variety of phagocytic receptors, including TAM, BAI1, stabilin-2, αVβ5 integrin, into a downstream of cytoskeletal responses 49. For example, BAI1receptor forms a complex with ELMO and DOCK180 to activate Rac1 GTPase 50. αVβ5 integrin can also initiate signaling through the p130Cas-CRK-DOCK180 axis 42. Meanwhile, MerTK and stabilin-2 can facilitate the activation of Rac1 through an adaptor protein called GULP 42. Once activated, Rac1 establishes an interaction with the WAVE regulatory complex, which triggers an activation of the Arp2/3 complex 13, which in turn enables the formation of branched actin filaments. These branching networks cause an outward displacement of the plasma membrane, thereby facilitating the emergence of pseudopods and the subsequent envelopment of the apoptotic cell 13. Rac1 signaling is a key regulator of cytoskeletal rearrangement during efferocytosis, and its activation is required for efficient apoptotic cell engulfment 51. Disruption of efferocytic pathways has been shown to impair clearance of apoptotic cardiomyocytes following myocardial infarction, leading to delayed resolution of inflammation and adverse ventricular remodeling 52.

***RhoA-Mediated Contraction Control.*** In contrast to Rac1, RhoA acts as a negative regulatory influence in such a way that it is upregulated during the final stages of engulfment to prevent overly rapid internalization 53. RhoA activates ROCK (Rho associated coiled-coil kinase), which in turn phosphorylates myosin light chain (MLC), thereby causing the contraction of actomyosin. This mediation controls the timing of the cup closure, helping to prevent inappropriate or excessive engulfment in the tissues. However, prolonged activation of RhoA can inhibit pseudopod extension and impair efferocytosis, which makes it very important for it to be tightly regulated to ensure the effective phagocytosis of apoptotic cells 53.

***Cdc42 and Pseudopodia Formation.*** Another important GTPase that is important in the engulfment of apoptotic cells is Cdc42. This protein helps to shape the cell membrane by forming small protrusions called filopodia, which eventually assist in sensing, contacting and engulfing apoptotic cells. Cdc42 activates the WASP/N-WASP complex, which in turn works through the Arp2/3 complex to promote actin filament branching which is an essential step for forming the cytoskeletal extensions that is needed to reach the dying cell 50, 53. These finely regulated GTPase-dependent signaling pathways act to carefully control the cytoskeletal reorganization necessary for the engulfment of apoptotic cells 50, 53. The fine balance between Rac1 mediated actin polymerization and RhoA induced contractions and closure enables precise phagocytosis 53, 54. Meanwhile, Cdc42 supports the stabilization of cytoskeletal structures and directional movement of the phagocyte 13, 42. Disruptions to these signaling pathways could compromise the removal of apoptotic cells and contribute to inadequate resolution of inflammation, as observed in chronic cardiovascular and autoimmune disorders 11, 45, 48.

**Figure 3:**
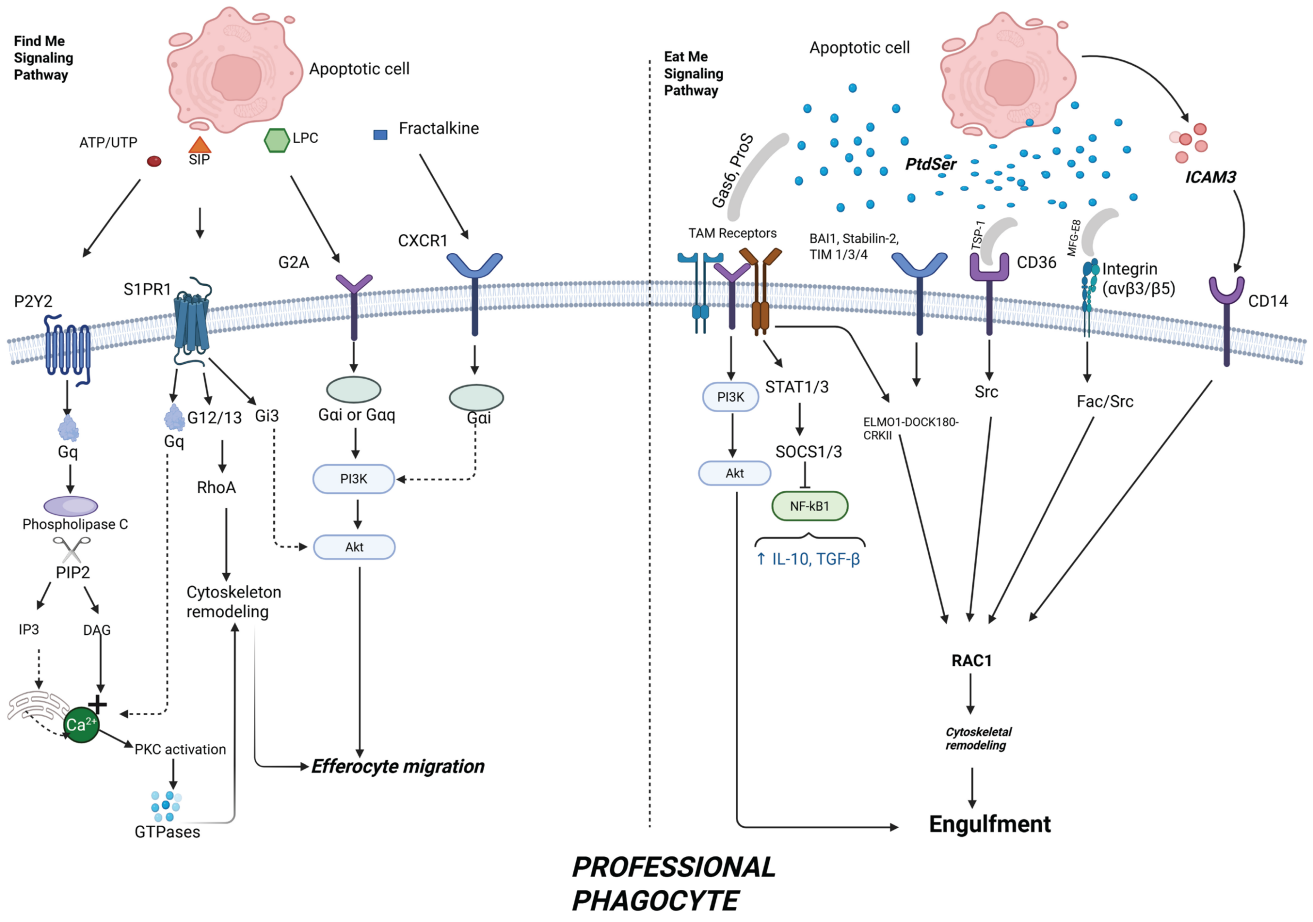
** Cellular mechanisms of apoptotic cell engulfment and degradation during efferocytosis.** This diagram shows the internal phases of efferocytosis. In step 3, efferocytes initiate engulfment through receptor signaling (e.g., TAM receptors, BAI1, Stabilin-2), activating intracellular pathways involving ELMO1–DOCK180 and RAC1 to drive cytoskeletal rearrangement. In step 4, phagosome maturation involves recruitment of Rab5 and Rab7, and fusion with lysosomes to form phagolysosomes. LC3-associated phagocytosis (LAP) supports this degradation process, with the involvement of lysosomal proteins such as LAMP1/2. These steps ensure safe breakdown of apoptotic cell contents and prevent inflammatory responses. Created with BioRender.com.

**Intracellular Degradation of Apoptotic Cells.** After the engulfment of apoptotic cells, the next important step is to degrade the dead cells internally (**Figure 3**). This step not only ensures the elimination of cellular waste but also enables the reprogramming of phagocytes into anti-inflammatory phenotypes which ultimately contributes to the maintenance of tissue equilibrium 28. Upon being engulfed internally, the phagosome undergoes a series of changes, where it merges with early and late endosomes before finally integrating with the lysosomes to form phagolysosomes 42. This fusion activates the degradation of the apoptotic cells through the action of hydrolytic enzymes, low pH, and reactive oxygen species (ROS) 13, 42. Several molecular components regulate this trafficking:

- Rab5 and Rab7 GTPases direct the maturation of phagosomes from early to late stage and also facilitate the fusion process with lysosomal compartments 55.
- LAMP1 and LAMP 2 proteins are upregulated, thereby promoting the process of lysosomal formation and maintaining phagolysosomal integrity 56.

Another unique process called LC3-associated phagocytosis (LAP) also plays an important role in the degradation of apoptotic cells, and is drawing some attention 57. In contrast to standard autophagic processes, LAP involves the recruitment of LC3-II to single-membrane phagosomes, which boosts its ability to fuse with lysosomes and speeds up the degradation process 57. This process is regulated by a complex network that consists of molecules like Rubicon, NOX2, and autophagy-associated proteins such as ATG5 and Beclin-1, which bring about the production of reactive oxygen species (ROS) 57.

Beyond simple digestion, apoptotic cell degradation is connected with the phenotypic behavior of the phagocyte in terms of anti-inflammatory signaling pathways. As the phagocyte degrades its content, it is accompanied by the activation of nuclear receptors such as PPARγ and LXRα, which drive the transcription of anti-inflammatory cytokines such as IL-10 and TGF-β and repress pro-inflammatory signals such as NF-κB 45. This modification ensures immune tolerance by preventing the release of intracellular danger signals. When there is incomplete or delayed phagocytic degradation, it can result in phagosome overload, which can consequently impair the process of efferocytosis, causing chronic inflammatory responses and secondary necrosis of engulfed apoptotic cells. Poor or incomplete degradation has been linked to chronic inflammatory conditions such as HF, atherosclerosis, and autoimmune disorders 45.

***Role of Phagocytic Receptors: Focus on the TAM Family (Tyro3, Axl, MerTK).*** Although, there are a number of cell receptors that are important for recognition and clearance of apoptotic cells, the TAM family of receptors which consist of Tyro3, Axl and MerTK play critical roles in the process of efferocytosis 42. These receptor tyrosine kinases are majorly expressed on professional phagocytic cells such as macrophages and dendritic cells and are activated through their interactions with bridging molecules like Gas6 and Protein S, which are usually bound to the residues of PtdSer that are normally exposed on the surface of apoptotic cells 42. TAM receptors fail to directly recognize apoptotic cells and, as a result, use the previously mentioned bridging molecules as a bridging system rather than mechanisms of direct recognition 58. The two primary bridging ligands, Gas6 and Protein S are the most widely researched ligands for the TAM receptors and function like opsonins by simultaneously binding PtdSer on apoptotic cells and the extracellular domains of TAM receptors on the professional phagocytes 58. The binding of these ligands to their corresponding receptors triggers a series of intracellular signaling events that are very important for rearranging the cytoskeleton and more importantly, for the engulfing process 58. Some other ligands like Gelectin-3 and tubby-like protein 1 have also been suggested to activate TAM family of receptors in efferocytosis. It is also important to note that among the TAM receptors family, MerTK has been associated with enhanced efferocytosis efficiency, particularly within tissue resident macrophages and in inflammatory contexts 58.

The activation of TAM receptors does not only play a key role in cellular engulfment but is also critical roles in the activation of transcriptional pathways that eventually leads to the resolution of inflammation. This is carried out through the production of anti-inflammatory cytokines such as TGF-β and IL-10, especially after the activation of MerTK and Axl in particular, while at the same time downregulating the production of pro-inflammatory cytokines such as TNF-α and IL-12 58. MerTK activation also triggers the activation of nuclear receptors PPARγ and LXRα, which help to reprogram phagocytes towards an anti-inflammatory phenotype and regulate lipid metabolism which are processes crucial for maintaining balance after efferocytosis 59. In the context of HF, activation of MerTK is of critical importance because the post-injury myocardium represents an immunologically active tissue, where deficiency in efferocytosis can trigger a maladaptive inflammatory cascade, resulting in ventricular dysfunction and fibrosis 15. Recent findings have suggested that the deficiency or inactivation of MerTK leads to the deficient clearance of apoptotic cardiomyocytes, thereby making neutrophilic inflammation worse, increasing fibrosis and, worsening cardiac function in the aftermath of an ischemic injury 15. While MerTK is constantly expressed in phagocytes, Axl tends to be expressed only when macrophages are activated in response to infections or periods of oxidative stress. Axl has also been commonly linked to dendritic cells during viral infections, although it is also involved in the maintenance of vascular homeostasis and fibrotic remodeling. It is also important to note that its expression is typically upregulated in conditions of chronic inflammation or oxidative stress, which are often observed in advanced HF, and may help promote immune tolerance in chronic disease states 58.

Among the TAM family of receptors, Tyro3 is the least characterized. Notably, the hematopoietic system displays a wide-ranging expression of Tyro3, with prevalence in monocytes and macrophages, dendritic cells, natural killer (NK) cells, platelet and megakaryocytes, and osteoclasts 60. Its specific role in the cardiac microenvironment specifics remains unclear and needs further research. Altogether, TAM family of receptors do not only bridge the gap between apoptotic cell recognition and immune resolution but also function as immunomodulatory checkpoints 60. In the context of heart failure, the dysregulated expression of these receptors, which can arise due to mechanisms including cleavage, insufficient ligands, and chronic inflammatory signaling, presents a potential therapeutic target for the restoration of immune equilibrium and the promotion of tissue repair 15.

**Immunomodulatory Signaling Molecules in Efferocytosis.** During the highly coordinated molecular process of efferocytosis, there are a number of signaling molecules that do not only help in the clearance of apoptotic cells (as discussed earlier) but also play critical roles in the resolution of inflammation and tissue repair. Among these molecules are the bridging molecules Gas6 and Protein S, as well as anti-inflammatory cytokines such as TGF-β and IL-10, which all play key regulators roles 15. Recently, a unique category of signaling lipids known as specialized pro-resolving mediators (SPMs) has also emerged as very important contributors to the resolution of inflammation and the promotion of corrective efferocytosis 45. These mediators, derived from omega-3 and omega-6 fatty acids, orchestrate the transition from inflammation to resolution by modulating phagocyte function and cytokine production 45. In the context of atherosclerosis and heart failure, where chronic inflammation and impaired resolution are central features, these immunomodulatory pathways are increasingly recognized as critical determinants of cardiac remodeling and functional recovery 45.

***Gas6 and Protein S: Bridging Molecules and TAM Receptor Activators.*** Gas6 and Protein S are a set of vitamin k-dependent proteins that play roles as bridging molecules by binding to the TAM family of receptors to activate them while simultaneously binding to PtdSer residues on apoptotic cells. Their dual-binding ability ensures specific and efficient efferocytosis 58, 61. Among these bridging molecules, Gas6 shows a high binding affinity for Axl and MerTK while, Protein S prefers to activate Tyro3 and MerTK depending on the cellular context 58. The binding of these ligands activates a signaling pathway of anti-inflammatory responses that involves the activation of Rac1, reorganization of the cytoskeleton and the transcriptional upregulation of genes encoding IL-10 and SOCS1/3 which suppresses pro-inflammatory signaling 61. In models of HF, research findings have indicated that the Gas6-MerTK signaling pathway provides cardioprotective benefits, by enhancing the clearance of apoptotic cardiomyocytes and thereby reducing adverse remodeling processes of the heart following myocardial injury 15. Additionally, in a recent study, it was observed that an overexpression of Gas6 in myocardial injury murine models greatly improved cardiac dysfunction 62. On the contrary, evidence from a study indicated that a reduction in MerTK activity through gene deletion or enzymatic cleavage impairs the process of resolving inflammation and instead worsens it, thereby highlighting the importance of MerTK signaling pathway in cardiac repair processes 42.

***TGF-β and IL-10: Immunosuppressive Cytokines in Post-Efferocytic Reprogramming.*** After phagocytes engulf apoptotic cells, macrophages in particular undergo a reprogramming in their phenotype to M2 which is characterized by the production and release of anti-inflammatory cytokines like TGF-β and IL-10 which are important to resolving inflammations and the repair of tissues 63. TGF-β helps in the activation of fibroblasts and the reorganization of the extracellular matrix, while also directing macrophage polarization toward the healing M2 phenotype. However, when there is unchecked or persistent TGF-β activity, then it may also contribute to the development of pathological fibrosis which can worsen HF if not properly regulated 64. IL-10 on the other hand blocks inflammation via the STAT3 signaling pathway by suppressing NF-κB activation, thereby inhibiting the production of pro-inflammatory cytokines such as TNF-α and IL-6 64. In cardiac contexts, the production of IL-10 after efferocytosis has been linked with improved inflammatory resolution, smaller heart injuries, and improved heart function following infarction in experimental models, hereby underscoring its role as a pivotal effector molecule in the transition of immune response from clearance to repair 11.

***SPMs: Lipid-Derived Inflammation Resolvers.*** When the resolution of inflammation is in progress, the metabolism of omega-3 and omega-6 fatty acids produces a set of SPMs which comprise of resolvins, lipoxins, protectins, and maresins 51. These molecules promote M2 macrophage polarization, limit the infiltration of neutrophils, and thereby enhance efferocytosis 45, 51. Among them, Resolvin D1 and Maresin 1 has been found to be very efficient in improving efferocytosis within cardiovascular models by enhancing the expression of MerTK and accelerating the maturation of the phagosome in phagocytes 15. In a murine model study 65, it was observed that increased levels of Resolvin D1 enhanced macrophage clearance in areas of infarction, thereby showing the importance of Resolvin D1 in promoting the resolution of inflammation. SPMs also complement cytokines like IL-10 to further support anti-inflammatory signaling while also reducing lipid peroxidation and oxidative stress within the cardiac tissue compromised by injury 66, 67. Furthermore, SPMs activate specific G-protein-coupled receptors like ALX/FPR2, ChemR23, and GPR32, on phagocytes and endothelial cells which enhances efferocytosis and restoration of vascular integrity 68, 69. Their role in resolving cardiovascular inflammation has presented promising novel therapeutic options, particularly in response to myocardial infarction where defective resolution of inflammation can contribute to the progression of HF. While these signaling pathways govern the recognition and engulfment of apoptotic cells, emerging evidence indicates that intracellular metabolic reprogramming plays a critical role in regulating the efficiency and sustainability of efferocytosis 70.

***Metabolic Regulation of Efferocytosis.*** Efferocytosis is closely linked to macrophage metabolic reprogramming that regulates cardiac inflammatory and pro-resolving functions 71. Following apoptotic cell uptake, macrophages undergo metabolic shifts that support sustained efferocytosis and resolution of inflammation 71. For instance, arginine metabolism has emerged as a key pathway, where increased arginase-1 activity promotes Rac1-dependent cytoskeletal rearrangement and enhances subsequent efferocytic capacity 72. Additionally, metabolic reprogramming suppresses pro-inflammatory cytokine production, facilitating a transition toward a pro-resolving phenotype 70. Mitochondrial function also plays a central role in sustaining efferocytosis, as efficient oxidative metabolism supports continued apoptotic cell clearance 73. A recent study by Liebold *et al.* indicated that the identity of apoptotic cells may contribute to the establishment of specific IL4-mediated immune programs in macrophages 74. Another study by A-Gonzalez *et al.* suggested that phagocytosis is a source of macrophage heterogeneity and acts synergistically with tissue-derived factors to maintain homeostasis 75. This niche-dependent macrophage programming ensures macrophages perform specialized roles that is adapted to their microenvironment rather than a uniform role in immune response.

In the context of HFpEF, where metabolic dysfunction is a defining feature 76, impaired macrophage metabolism may contribute to defective efferocytosis and persistent inflammation. The identity of apoptotic cells also plays a critical role in shaping macrophage programming following efferocytosis. Emerging evidence demonstrates that macrophages adopt distinct functional phenotypes depending on the origin and molecular composition of engulfed cells 76. This niche-dependent programming influences inflammatory signaling, metabolic activity, and subsequent efferocytic capacity 76. In the myocardium, clearance of apoptotic cardiomyocytes versus other cell types may differentially regulate macrophage behavior, thereby influencing inflammation resolution, tissue repair, and cardiac remodeling outcomes 76. These findings highlight an additional layer of complexity in efferocytosis, where the nature of the apoptotic cargo actively determines downstream immune responses.

## Dysregulation of Efferocytosis in Heart Failure

In HF, cardiomyocytes normally undergo apoptosis as a result of conditions that include ischemia, mechanical overload, and metabolic dysfunction, which then initiates the inflammatory response that contributes to HF 48. In a normal heart condition, these dead cells are rapidly cleared by efferocytosis to prevent secondary necrosis and promote the resolution of inflammation. However, in the failing heart tissue, the process of efferocytosis becomes impaired, which then leads to the accumulation of dead cells that ultimately leads to chronic inflammation, fibrosis, and worsening of cardiac function 11. Over time, this accumulation of dead cells can eventually lead to the formation of plaques and worsen the HF condition 11. So why does efferocytosis fail in cases like advanced HF? Macrophages, which are the major phagocyte players in efferocytosis, play a central role in impaired efferocytosis. A couple of molecular mechanisms associated with macrophages have been identified that can significantly reduce their clearance capacity in the failing heart or improve it. One of such molecular mechanisms, according to a research study, is the activation of a mechanosensitive ion channel called Piezo1, which hampers the efferocytosis process 77. It was also demonstrated by a research group that the expression of Piezo1 is upregulated in macrophages within the infarcted myocardium, and its activation with Yoda1; which is a chemical compound that activates Piezo1, reduces the capacity of macrophages to carry out efferocytosis in both *in vivo* and *in vitro* condition 77. They also observed that when Piezo1 is genetically deleted in macrophages, it significantly improved dead cell clearance and a much better remodeling of the ventricles after myocardial infarction (MI) 77, which signifies the important regulatory role that Piezo1 plays in impaired efferocytosis after cardiac injury.

On the other hand, Lipocalin 10 (Lcn-10) has been observed to be an important positive regulator of macrophage efferocytosis, especially after the event of a myocardial ischemia/reperfusion injury 78. In a research study using a murine model, it was found that mice deficient in Lcn-10 showed reduced engulfment of dead cells, and an increased accumulation of apoptotic cardiomyocytes, which made the heart function worse compared to the controls 78. This seems to suggest that the expression of Lcn-10 appeared to influence the expression of important efferocytosis-related genes, and may also contain a PtdSer binding motif that is necessary for recognizing apoptotic cells 78. The authors reported that restoring Lcn10 expression improved efferocytosis in the murine models, therefore making it a potential therapeutic target in cardiac repair 78. In the same manner, Legumain (Lgmn), which is a lysosomal protease highly expressed in macrophages, has been found to impair efferocytosis when it is deficient or missing 32. In a study 32, it was found out that Lgmn deficiency led to a worsened cardiac function as a result of the accumulation of dead cardiomyocytes, defective phagolysosomal degradation, and the excessive recruitment of inflammatory monocytes and macrophages. Additionally, the authors also found that a deficiency in Lgmn suppressed the production of anti-inflammatory cytokines such as IL-10 and TGF-β, while enhancing the production of pro-inflammatory cytokines like IL-1β, TNF-α, IL-6 and IFN-γ that all promote chronic inflammation and heart tissue injury 32. Additionally, researchers have directly linked the dysfunction of the phagocytic receptor MerTK with impaired efferocytosis and maladaptive remodeling of the heart following an infarction 79. It was observed in a study 79, that mice models lacking MerTK showed a significant accumulation of apoptotic cardiomyocytes with reduced efferocytosis in vivo and delay in resolving inflammation. This did not only impair phagocytic clearance but also directly increased infarct size and adverse cardiac remodeling. More importantly, according to the bone marrow transplantation experiments carried in the same study 79, they found that restoring MerTk’s function in bone marrow-derived cells revived systolic function, thereby signifying the essential role that MerTK plays in carrying out effective efferocytosis and heart repair.

**Figure 4:**
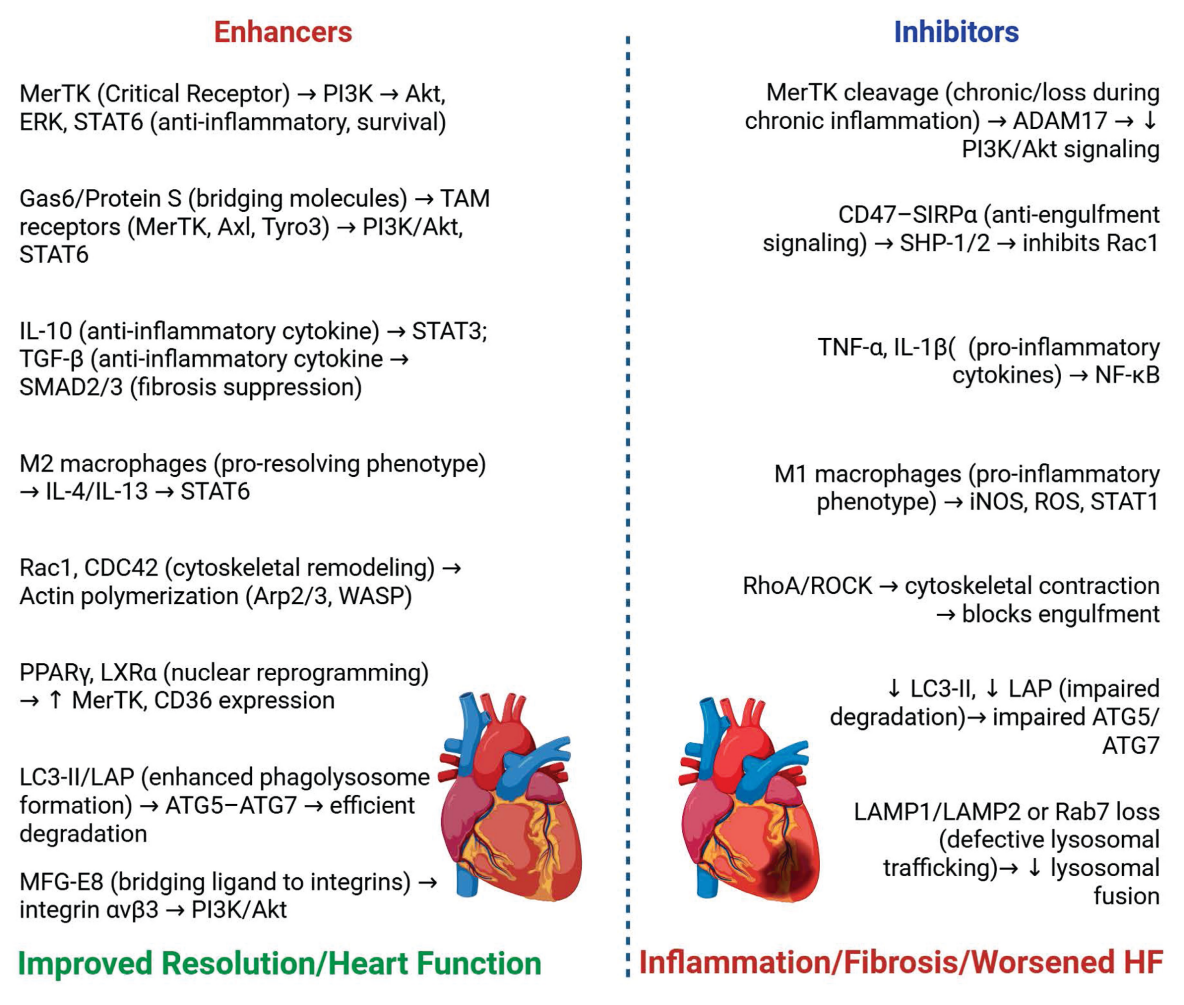
** TAM receptor signals healthy health and failing heart.** This schematic differentiates TAM receptor activity in healthy versus failing heart. In a healthy heart, MerTK and other TAM receptors promote apoptotic cell clearance, inhibit NF-κB, and stimulate IL-10 and TGF-β production—leading to inflammation resolution and cardiac tissue repair. In heart failure, MerTK is cleaved, CD47–SIRPα signaling blocks efferocytosis, and PI3K/Akt and SOCS1/3 signaling are diminished. These changes promote persistent inflammation, fibrosis, and progressive cardiac dysfunction. Created with BioRender.com.

In addition to TAM receptor–mediated pathways, other scavenger receptors have been shown to contribute to efferocytosis and cardiac remodeling. Macrophage scavenger receptor A (SR-A) has been suggested to regulate post-infarction inflammation, where its deficiency leads to increased TNF-α expression, enhanced MMP9 activity, and increased susceptibility to cardiac rupture 80. Furthermore, MMP9 has been implicated in modulating efferocytosis through CD36-dependent pathways, where its depletion enhances macrophage-mediated clearance of apoptotic cardiomyocytes and reduces adverse remodeling 81. These findings highlight the interplay between extracellular matrix remodeling and efferocytosis in heart failure progression. It has increasingly become recognized in clinical settings that impaired efferocytosis is a major contributor to advanced HF 51, 52. Emerging biomarkers like circulating apoptotic endothelial microparticles may be able to predict clearance capacity, particularly in HFpEF 11. Therapeutically, there are emerging approaches like enhancing macrophage function, engineering chimeric antigen receptor macrophages (CAR-M) therapies, and targeting lysosomal degradation pathways that are offering promising directions to restore impaired efferocytosis and improve the failing heart 82. This signaling is summarized in **Figure 4**.

**Chronic Inflammation, Fibrosis, and Inflammatory Mediators in Cardiac Remodeling.** The continuous accumulation of dead cardiomyocytes in the heart as a result of impaired efferocytosis sustains chronic inflammation within the myocardium, which eventually leads to and promotes substantial maladaptive tissue responses 45. Some of these tissue responses are the activation of fibroblasts, deposition of extracellular matrix, and excessive fibrosis, all as a result of prolonged exposure to unresolved inflammation, which eventually leads to stiffening of the ventricles and impaired cardiac contractility 45. In addition to pro-inflammatory cytokines like TNF-α and IL-6, recent studies have also identified the NLRP3 inflammasome as a major player in HF. Activation of the NLRP3 inflammasome triggers a downstream production of IL-1β and IL-18, which have been shown to further promote myocardial injury, ventricular remodeling, and more importantly, worsening HF condition 83. This ensures a constant cycle in which dysregulated efferocytosis not only promotes chronic inflammation but also directly contributes to the structural and functional deterioration of the failing heart in HF.

## Therapeutic Targeting of Efferocytosis in Heart Failure

**Figure 5:**
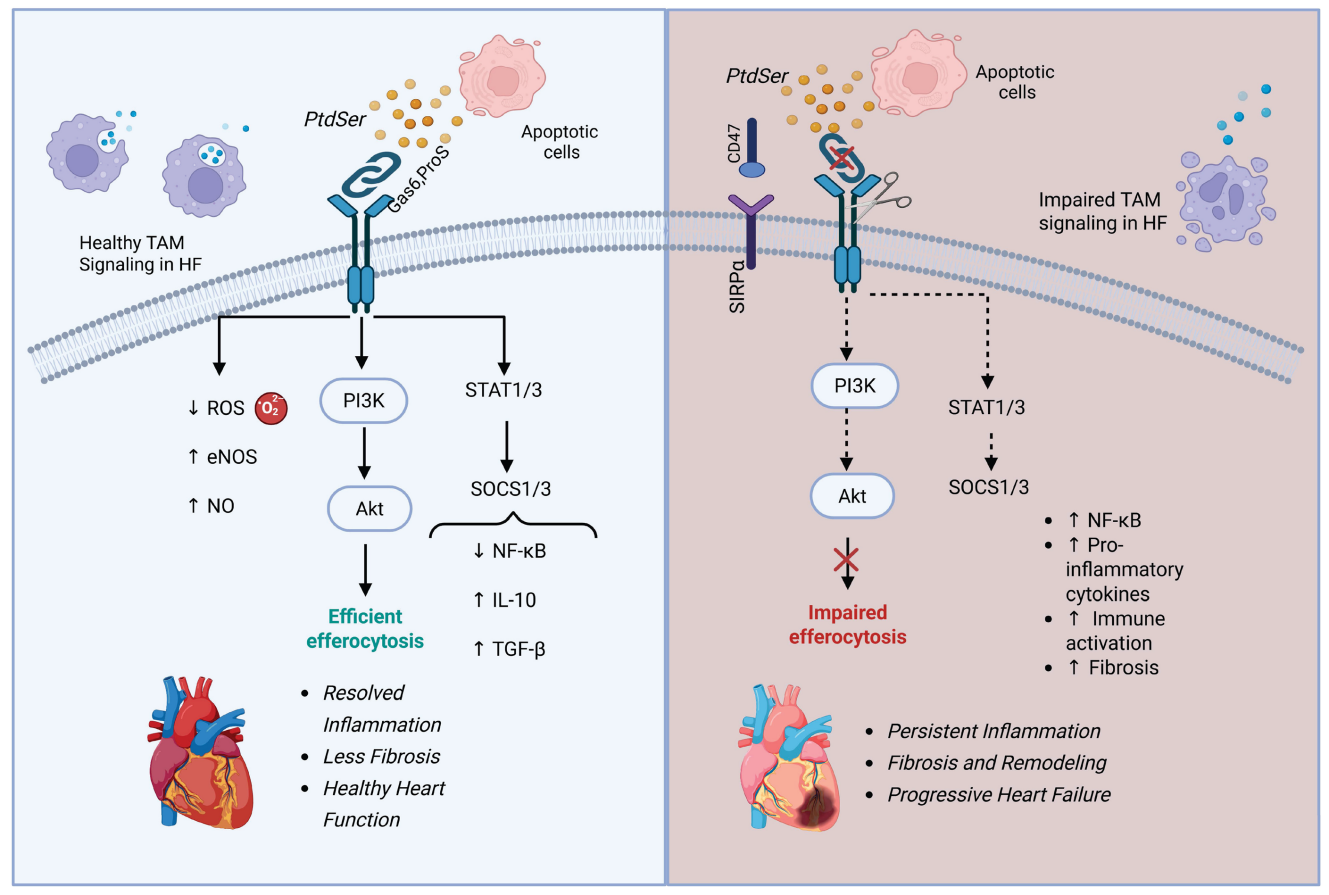
** Key molecular and cellular factors modulating efferocytosis and their impact on heart failure.** Enhancing factors (e.g., MerTK, IL-10, M2 macrophages) promote apoptotic cell clearance, immune resolution, and improved cardiac outcomes. Inhibitory factors (e.g., CD47–SIRPα, TNF-α, M1 macrophages) impair efferocytosis, leading to unresolved inflammation and worsened heart failure. The figure illustrates how the balance of these signals influences disease progression. Created with BioRender.com.

**Strategies to Enhance Efferocytosis.** Impaired efferocytosis contributes greatly to the worsening of HF condition, and as such, multiple therapeutic approaches have been proposed to enhance and restore proper apoptotic cardiomyocyte clearance in the failing heart. A couple of these strategies target the recognition, engulfment, and degradation of these cells, as well as the phenotypic reprogramming of major phagocyte players to an anti-inflammatory type (**Figure 5**).

***Enhancing MerTK Activation and TAM Signaling.*** Given the important role of MerTK in efferocytosis and the resolution of inflammation, there have been a couple of recent studies targeting this receptor that have emerged as promising avenues for HF management 84 85. The activation of MerTK is essential because it does not only facilitate the clearance of dead cardiomyocytes but also, more importantly, plays a key role in the resolution of inflammation through the downstream activation of nuclear receptors such as PPARγ and LXRα, which are both crucial for lipid homeostasis and the reprogramming process of macrophages to an anti-inflammatory type 59, 64. Preclinical studies such have also shown that deficiency of MerTK receptors on phagocytes leads to an impaired clearance of cardiomyocytes, neutrophilic infiltration, myocardial fibrosis, and worsening heart condition after an ischemic injury, which could all result in progression of HF 15.

Gas 6, which is a bridging molecule for TAM receptors, has also emerged as a key therapeutic target via the Gas6-MerTk signaling axis 62. Recent studies *in vivo* and *in vitro* models have demonstrated that overexpression of Gas6 improved efferocytosis and, thereby, cardiac function following myocardial infarction, while on the contrary, deficiency led to decreased efferocytosis 62. Although, MerTK remains the most widely studied receptor of the TAM family, both Axl and Tyro3 still contribute importantly to dead cardiomyocyte clearance and immune regulation, particularly during specific pathological conditions 62. The expression of Axl is typically upregulated during conditions such as oxidative stress, viral infections, and chronic inflammation in a bid to dampen excessive immune responses 58. In the failing heart, an increase in oxidative stress may induce the expression of Axl, which suggests that it serves as a compensation mechanism to help limit chronic inflammation during the advanced stages of HF. Tyro3, which hasn’t been studied enough in the context of the cardiac microenvironment is expressed across multiple immune cells populations including macrophages, dendritic cells, natural killer cells, and platelets 60. While the direct role of Tyro3 in myocardial efferocytosis remains unclear, it’s broad distribution among immune cell populations strongly suggests that it may be a strong participant in immune homeostasis and vascular integrity, which is important for proper cardiac function. Altogether, TAM receptor signaling represents an elaborate axis for therapeutic targeting, where MerTK remains well studied, while Tyro3 and Axl present unexplored and promising areas for research in the context of efferocytosis in HF.

***SPMs as Pro-Efferocytic Agents.*** SPMs (including resolvins, protectins, lipoxins, and maresins) have shown strong efferocytosis and anti-inflammatory effects in various cardiovascular models 86. These lipids which are derived from omega-3 and omega-6 fatty acids enhance efferocytosis, limit neutrophil infiltration, as well as suppress oxidative stress and inflammatory cytokine release 45,66,67. Among these SPMs, Resolvin D1 and Maresin have been shown to upregulate the expression of MerTK whilst also increasing the maturation of the phagosome in phagocytes, which leads to an increase in efferocytosis following myocardial infarction 15. In a research study carried out in murine models, it was shown that an administration of Resolvin D1 improved macrophage clearance of dead cardiomyocytes whilst also enhancing the function of the left ventricle 65. Additionally, SPMs have also been shown to activate some specific G-protein-coupled receptors like ChemR23 and GPR32, to further enhance the process of efferocytosis in the failing heart 69.

***Potential Pharmacological Agents.*** As we continue to build on our understanding of efferocytosis in HF, several pharmacological agents are currently being explored to therapeutically target key pathways that are involved in apoptotic cell clearance. Many of these agents are aimed at targeting signaling pathways, immune regulatory pathways, and intracellular mechanisms that control the efficiency of efferocytosis. One promising target is the MerTK receptor pathway, which has an important role in efferocytosis of cardiomyocytes and the resolution of inflammation 84. Although, because of its diverse effects through either its activation or inhibition, most of the research on MerTK receptor pathway focuses on inhibiting its expression in the tumor microenvironment of cancer 45. Nevertheless, there are some insights into how enhancing MerTK activation is beneficial for efferocytosis and inflammation resolution. Recent studies have identified a couple of synthetic agonists, metabolites and natural compounds that can enhance the expression of MerTK and its downstream anti-inflammatory signaling 85. For example, in an *in vitro* study, it was demonstrated that cells treated with the natural compound Resveratrol showed increased efferocytosis 87, although the cells were thymocytes, which suggests that using the same in apoptotic cardiomyocytes might present same result. Another important pharmacological aspect involves the regulation of IL-10 signaling. For example, a recent *in vitro* and *in vivo* study explored a recombinant IL-10 therapy which showed its ability to suppress NF-κB activation, inhibit pro-inflammatory cytokine production and significantly improved the function of the heart after myocardial injury 88. Agents like this, aim to leverage the ability of IL-10 to reprogram macrophages to an anti-inflammatory phenotype that favors efferocytosis and tissue repair 51, 89. In the same manner, the modulation of TGF-β has also attracted interest because of its double role in tissue repair and fibrotic remodeling 11. Pharmacological agents such as TGF-β receptor blockers and a couple of fibrotic compounds are being evaluated for their ability to reduce maladaptive fibrosis while ensuring that they maintain their beneficial repair effects, which is necessary for the resolution of inflammation after efferocytosis 11.

Aside from cytokine-based therapies, there have been emerging research studies that have focused on targeting intracellular regulators such as ROCK (Rho-associated kinase), which play regulatory roles in the rearrangement of actin cytoskeleton during phagocytic cup formation. In a recent study, it was shown that inhibition of the Rho-ROCK signaling pathway enhanced efferocytosis by macrophages and also resolved inflammation in a murine model 90, which suggests that similar mechanisms could be therapeutically beneficial in HF. Additionally, there has also been an increasing interest in the pharmacological inhibition of the NLRP3 inflammasome because of the ability of this action to reduce inflammation, secondary necrosis, and cardiomyocyte apoptosis, which would indirectly improve efferocytosis 11. For example, research using NLRP3 knockout model showed that reduced IL-1β levels are associated with the reduction of cardiac atrophy and improved heart function in cardiac inflammatory conditions 91. Although, more research is needed to clarify the direct contribution of NLRP3 signaling in cardiomyocytes to efferocytic outcomes. Collectively, these evolving pharmacological approaches show promising avenues to restore the balance between apoptotic cardiomyocyte clearance, resolution of inflammation and effective cardiac repair.

***Role of Mesenchymal Stem Cells in Improving Apoptotic Cell Clearance.*** In recent years, Mesenchymal Stem Cells (MSCs) have drawn attention for their capacity to regulate immune responses and promote tissue repair in the failing heart 92. In addition to their ability to differentiate into various stromal cell types, and their well-known paracrine effects, MSCs also carry out non-professional phagocytosis which also includes the clearance of apoptotic cells, through mechanisms that resembles that of efferocytosis through the use of Axl and Tyro3 as their main receptors 93. Interestingly, MSCs infused in the heart using *in vivo* models also demonstrated their ability to indirectly influence and promote efferocytosis of cardiomyocytes by influencing macrophage polarization to the M2 type whilst also enhancing macrophage clearance 92. This auxiliary ability to directly or indirectly allows MSCs to support the resolution of inflammation and promote tissue repair in the heart. Several preclinical studies have also shown that extracellular vesicles derived from MSCs can transfer active molecules like anti-inflammatory cytokines, microRNAs, and lipids that enhance the rate of efferocytosis of apoptotic cardiomyocytes 94, 95. The combined paracrine functions and efferocytic ability of MSCs suggest that they hold significant promise as adjunct therapies for enhancing efferocytosis of cardiomyocytes in advanced HF.

***Druggable Targets in Efferocytosis.*** Efferocytosis represents a highly promising therapeutic target, with multiple druggable pathways across its distinct phases, including recognition, engulfment, and post-engulfment immunoreprogramming 51. Among these, the TAM receptor MerTK has emerged as a central regulator, and pharmacological modulation of this pathway is currently being explored in clinical settings 51. Small-molecule inhibitors targeting TAM signaling, such as MRX-2843, are under investigation in clinical trials (Meryx, Inc-NCT03510104 and NCT04872478; Emory University- NCT04762199), highlighting the translational potential of modulating efferocytic pathways. Beyond receptor targeting, recent studies have identified pharmacological agents capable of enhancing efferocytosis. Protocatechuic acid, a naturally occurring polyphenol found in fruits and vegetables, has been shown to improve macrophage efferocytic capacity and attenuate inflammatory responses 96. Similarly, the FDA-approved drug disulfiram has demonstrated the ability to enhance efferocytosis and reduce atherosclerotic burden through modulation of macrophage function 97. In addition, thiothixene has been reported to stimulate macrophage-mediated clearance of pathogenic cells by inducing arginase-1 expression and promoting sustained efferocytic activity 98. These emerging pharmacological strategies highlight the feasibility of targeting efferocytosis to restore immune resolution and limit pathological remodeling. In the context of heart failure, where impaired apoptotic cell clearance contributes to chronic inflammation and fibrosis, such approaches may offer novel therapeutic avenues to improve cardiac repair and functional outcomes.

**Figure 6:**
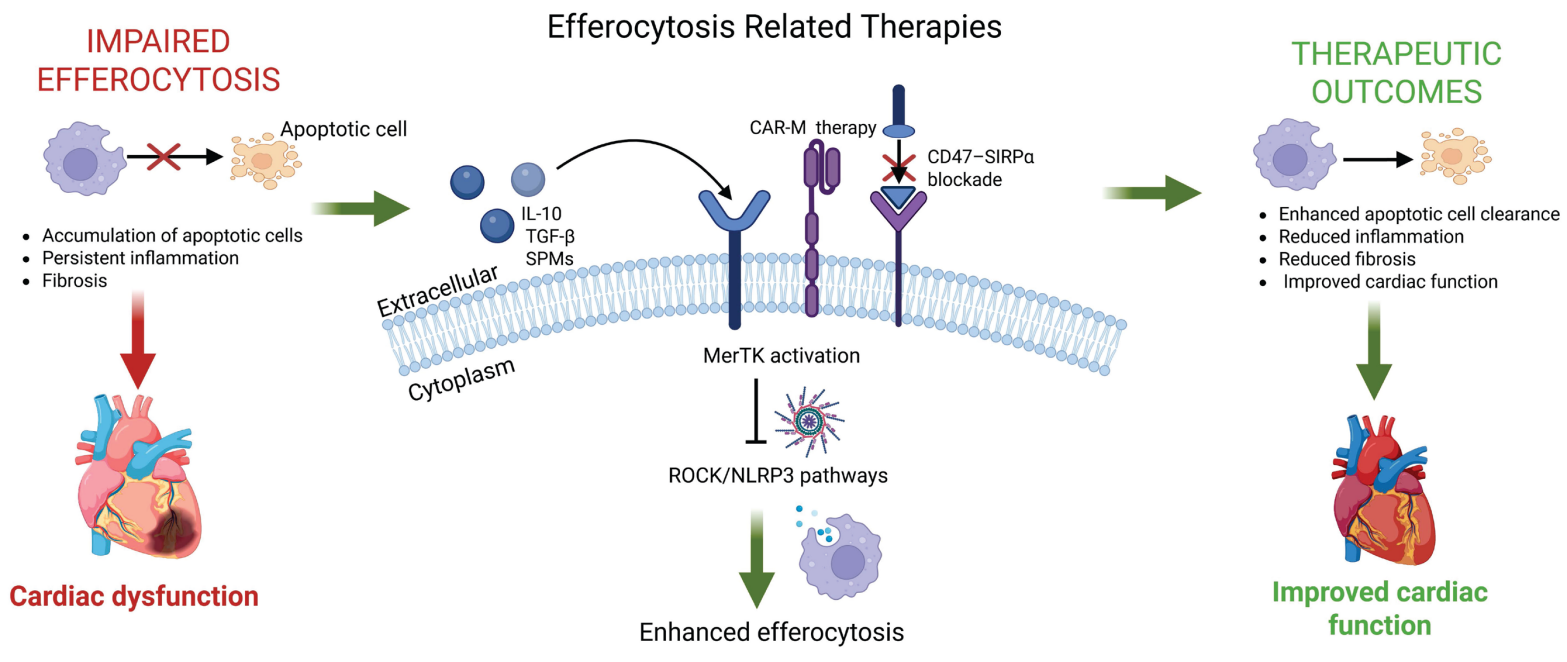
** Therapeutic strategies targeting efferocytosis and their impact on cardiac function.** Impaired efferocytosis leads to accumulation of apoptotic cardiomyocytes, persistent inflammation, and adverse cardiac remodeling. Therapeutic approaches (e.g., MerTK activation, CD47–SIRPα blockade, IL-10 and TGF-β signaling, SPMs, CAR-macrophage therapies, and inhibition of ROCK/NLRP3 pathways) enhance apoptotic cell clearance and promote inflammation resolution. These interventions collectively reduce fibrosis, improve cardiac remodeling, and enhance overall cardiac function. The figure illustrates how targeting efferocytosis pathways can shift the balance from chronic inflammation toward tissue repair and functional recovery. Created with BioRender.com.

***Preclinical and Clinical Evidence Supporting Efferocytosis-Targeted Therapies.*** Several preclinical research studies that are available have directly linked enhanced efferocytosis to improved cardiac outcomes in HF. For example, studies targeting the activation of MerTK have shown that restoring the function of this important receptor in *in vitro* and *in vivo* models not only improved apoptotic cardiomyocyte clearance, but also revived systolic performance after ischemic injury and atherosclerosis 79, 84. In the same manner, enhancing Gas6-MerTK signaling has also been shown to reduce inflammation, reduce infarct size and prevent against adverse remodeling 62. Emerging therapeutic approaches such as the use of CAR-M to modulate the immune microenvironment and enhance efferocytosis are now being explored in HF models. CAR-Ms in increasing evidence have shown their potential to restore impaired efferocytosis, reduce fibrosis, and promote favorable remodeling after myocardial infarction 99. Recent studies have demonstrated that CAR-M therapy can be engineered to recognize fibrotic or apoptotic targets, thereby promoting their clearance and reducing cardiac fibrosis 82. In preclinical studies, CAR-M–mediated enhancement of efferocytosis has been associated with improved tissue remodeling and functional recovery following myocardial injury 100, 101. These findings highlight the translational potential of engineered macrophages as a therapeutic platform in heart failure. Additionally, enhancing lysosomal degradation pathways (such as boosting legumain activity) or modulating the maturation of phagolysosome through LC3-associated phagocytosis are being tested in *in vivo* models and have shown promising results in supporting cardiac repair 32, 57. Clinically, biomarkers like circulating apoptotic endothelial microparticles (EMPs) could also serve as predictors of impaired efferocytosis and HF progression, especially in HFpEF 102. Finally, even though this evidence is mostly from preclinical studies, these data strongly suggest that improving efferocytosis could help patients with HF by reducing inflammation and maladaptive cardiac remodeling. These therapeutic strategies targeting efferocytosis in cardiac function are summarized in **Figure 6**.

## Future Directions and Conclusions

Despite substantial advances in understanding efferocytosis in HF, the field remains characterized by several critical knowledge gaps and unresolved questions. Importantly, current insights are largely derived from experimental models, and their translation to human heart failure remains incomplete. A more integrative understanding that links immune regulation, metabolism, and tissue remodeling is needed to fully elucidate the role of efferocytosis in disease progression and therapeutic response.

**Open Research Questions in Efferocytosis in Heart Failure.** HF seriously affects the lives of people living with the condition, and although significant progress has been made in understanding the role of efferocytosis in HF, many fundamental questions still remain unclear. The dynamic regulation of macrophage phenotypes, especially the transition between pro-inflammatory and pro-resolving during HF progression, requires further research 11. In particular, the precise mechanisms and different contributions of resident and recruited macrophages to dead cardiomyocyte clearance in HF also require further clarification 89. Moreover, MerTK from the TAM receptors is well studied, the contributions and intricate mechanisms associated with Tyro3 and Axl receptors in the context of cardiac efferocytosis and immune regulation are not well characterized 58, 103. It is also important to note that the effect of coexisting conditions like aging and diabetes on the function of cardiac efferocytosis have not been adequately explored yet 45. Additionally, the long-term effects and safety of prolonged efferocytosis-targeted therapies remain unclear, especially in the context of chronic HF 64. Finally, the development of proven biomarkers to assess the capacity of efferocytosis and guided targeted interventions in HF patients still remains an unmet need 102.

**Potential for Multi-Omics Integration to Identify Novel Therapeutic Targets.** There have been substantial recent advances in multi-omics technology, which offer tools that are needed to dissect complex pathways regulating efferocytosis in the heart. The integration of multi-omics could identify new therapeutic targets and biomarkers that are relevant to efferocytosis dysfunction in the failing heart 11. For instance, single-cell RNA sequencing studies have begun to define the heterogeneity of macrophages in cardiovascular disease 25. Proteomic profiling of TAM receptor pathways alongside post-translational modification studies could further make clear the regulatory mechanisms involved in impaired cardiac efferocytosis 11, 51. Together, these integrative approaches could speed up the discovery of advanced and next-generation efferocytosis therapies tailored to patient-specific immune and metabolic profiles.

**Translational Implications for Patient Management.** Targeting efferocytosis in the heart presents a novel therapeutic option with significant potential to complement existing HF treatments, because of its potential to reduce chronic inflammation, limit fibrosis, and improve the overall function of the heart 15, 52. Interventions like these could be of benefit to HFrEF and HFpEF patients whose condition coincides with impaired efferocytosis 102. Also, the development of biomarkers such as circulating endothelial microparticles (EMPs) could help health providers to distinguish patients that are most likely to benefit from efferocytosis-targeted therapies 25, 102. However, there are challenges that still remain in defining the optimum timing and dosing for these therapeutic therapies, as excessive stimulation of efferocytosis could lead to the risk of immunosuppression or even contribute to maladaptive remodeling of the heart 11.

**Summary of Key Takeaways and Research Gaps.** Efferocytosis is crucial for immune balance in the heart and impaired clearance of apoptotic cardiomyocytes is increasingly recognized as a major driver of HF progression. Several therapies aimed at enhancing efferocytosis through TAM receptor activation, IL-10 signaling, SPMs and CAR-M therapies have shown promise in HF. However, there are a few questions and areas for further research that we have identified.** 1**) What is the specific role of different macrophage populations (resident and monocyte-derived) in efferocytosis at various stages of HF?** 2**) While some studies have investigated Axl and Tyro3 signaling in certain disease contexts, what is their role in cardiac efferocytosis and how do they impact HF progression? **3**) How do coexisting conditions in patients like diabetes, obesity and aging affect cardiac efferocytosis, especially in advanced HF? **4**) We need biomarkers to predict efferocytosis capacity in HF patients. **5**) Finally, we need large clinical trials to test the safety of efferocytosis-targeted therapies in HF patients. As we move forward in HF research, we need to address these gaps and questions to translate findings into clinical therapies for HF patients.

## Figures and Tables

**Table 1:** ** Key “Find-Me”, “Eat-Me” and “Don’t Eat-Me” signals released by apoptotic cells** and their receptors on phagocytes, and supporting references. These signals all function together to establish a chemoattractant gradient that facilitates the efficiency and precise localization of apoptotic cell clearance. Created with BioRender.com.

Signal Type	Key Signals	Phagocyte Receptors / Partners	Description	References
Find-Me	LPC	G2A receptor	Lipid released via iPLA2 activation by caspase-3; acts as a chemoattractant.	Lauber *et al*., 2003
Find-Me	S1P	S1P receptor	Lipid mediator derived from sphingolipid metabolism that regulates the positioning of efferocytosis.	Gude *et al*., 2008
Find-Me	ATP / UTP	P2Y2 receptor	Nucleotides released from dying cells to recruit phagocytes via purinergic signaling.	Lamarca *et al*., 2014; Medina & Ravichandran, 2016
Find-Me	CX3CL1	CX3CR1 receptor	Membrane-bound chemokine cleaved during apoptosis to attract phagocytes.	Naessens *et al*., 2024
Eat-Me	PtdSer	Direct: BAI1, TIM-1/3/4, Stabilin-2, RAGE; Indirect: MerTK, Axl, Tyro3 via Gas6, Protein S, MFG-E8, TSP-1, integrins	Primary engulfment signal; flips to outer membrane during apoptosis; central to the process of efferocytosis	Naeini *et al*., 2020; Pinto *et al*., 2020
Eat-Me	ICAM-3	CD14	Facilitates direct apoptotic cell recognition	Medina & Ravichandran, 2016
Don't-Eat-Me	CD47	SIRPα	Inhibits engulfment via SHP-1/SHP-2 phosphatases, blocking cytoskeletal changes	Chen, 2024
Don't-Eat-Me	CD31	CD31–CD31 homophilic interaction	Prevents phagocytosis of viable cells; disrupted during apoptosis	Zhang *et al*., 2022
